# Piloting the “Anti-Oppressive Practice & Research with Diverse Older Adults” Training Program during COVID-19

**DOI:** 10.1093/geroni/igab046.3516

**Published:** 2021-12-17

**Authors:** David Camacho, Kelly Pacheco, Sabrina Feldman, Usha Kaul, Patricia Kim, M Carrington Reid

**Affiliations:** 1 University of Maryland Baltimore, Baltimore, Maryland, United States; 2 New York University, New York University, New York, United States; 3 Weill Cornell Medicine, Weill Cornell Medicine, New York, United States; 4 Weill Cornell Medical College, New York, New York, United States

## Abstract

There is a critical need to develop our gerontological-sensitive workforce. Social workers (SW) frequently provide services to older adults yet there are few opportunities for them to train as gerontological clinicians and/or researchers. To provide an opportunity for SW students to gain gerontological knowledge, clinical practice, and research skills, we developed, and pilot tested the “Anti-Oppressive Practice and Research with Diverse Older Adults” virtual training program at a major medical facility in Manhattan, NY. We explored the feasibility, implementation, and impact of this novel program. The 21-hour weekly MSW field placement program followed an anti-oppressive framework and included: 1) supervision and training sessions; and 2) direct clinical and research practice (e.g., theory, funding, assessment, data collection etc.) and aging topical seminars (e.g., depression, loneliness, pain etc.). Racially diverse supervisors and graduate SW students engaged in reflective writing exercises, iterative discussions (recorded & transcribed) and a thematic analysis of data. All interns successfully completed the program and reported enhanced skills related to SW core competencies and research (e.g., standardized assessments), research and practice gap awareness (e.g., minority aging) and plans to pursue advanced research training and/or gerontological clinical work. Intern challenges included: 1) disconnect between MSW curricula and research placements; and 2) managing minority and contextual stressors (e.g., imposter syndrome, covid-19, civil unrest). Supervisory challenges included: 1) humanizing sensitive discussions via virtual communication and 2) resource constraints. Future research should systematically assess program effects (e.g., SW core competencies) and how to facilitate interprofessional collaborations to develop diverse gerontological SWs and researchers.

